# Development and evaluation of an electronic algorithm using a combination of a two-step malaria RDT and other rapid diagnostic tools for the management of febrile illness in children under 5 attending outpatient facilities in Burkina Faso

**DOI:** 10.1186/s13063-022-06717-8

**Published:** 2022-09-15

**Authors:** Francois Kiemde, Adelaide Compaore, Fla Koueta, Athanase M. Some, Berenger Kabore, Daniel Valia, Toussaint Rouamba, Fadima Yaya Bocoum, Seydou Sawadogo, Macaire Nana, Diane Y. Some, Nadine A. Kone, Valentin Pagbeleguem, Inoussa Sangare, Antonia W. Bere, Massa dit Achille Bonko, Gautier Tougri, Sylvie Yeri Youl, Henk Schallig, Halidou Tinto

**Affiliations:** 1 Institut de Recherche en Sciences de la Santé – Clinical Research Unit of Nanoro (IRSS-CRUN), Nanoro, Burkina Faso; 2 Department of Pediatrics – CHU Yalgado Ouedraogo, Ouagadougou, Burkina Faso; 3 Health District of Nanoro, Ministry of Health, Nanoro, Burkina Faso; 4 National Malaria Control Program, Ministry of Health, Ouagadougou, Burkina Faso; 5 National Agency for Primary Healthcare, Ministry of Health, Ouagadougou, Burkina Faso; 6 Amsterdam University Medical Centers, Academic Medical Centre at the University of Amsterdam, Department of Medical Microbiology and Infection Prevention, Laboratory for Experimental Parasitology, Amsterdam Institute for Infection and Immunity, Meibergdreef 9, Amsterdam, 1105 AZ The Netherlands

**Keywords:** Fever, Rapid diagnostic test, e-Algorithm, Prescription, Artificial intelligence

## Abstract

**Background:**

In Sub-Saharan Africa (SSA), febrile illnesses remain a major public health problem in children. However, the persistence of *hrp2* antigen and the low sensitivity of *p*LDH RDT negatively affect antimalarials and antibiotics prescription practices. These limitations lead to poor management of febrile diseases and antimicrobial resistance (AMR). To improve the diagnosis of these febrile diseases and subsequent prescription of antimicrobials, it is hypothesized that the implementation of an algorithm including a two-step malaria RDT *Pf*HRP2/*p*LDH supported by point-of-care (PoC) tests for bacterial infections could significantly improve the management of febrile diseases and thereby tackling AMR.

**Methods:**

To assess the value of the proposed algorithm, an open-label randomized controlled trial with three arms, enrolling febrile children from 6 to 59 months is proposed.

In the control arm, febrile children will be managed according to the Integrated Management of Childhood Illnesses (IMCI), which is part of the standard of care in Burkina Faso. Treatment will be done according to national guidelines.

In the RDT decisional algorithm (RDT-DA) arm (intervention), the clinical examination based on IMIC will be supported by a two-step malaria RDT and bacterial infections RDTs. Prescription will be left to the discretion of the healthcare workers based on clinical examination and PoC test results.

In the e-algorithm arm (intervention), artificial intelligence integrating multiple layers of clinical information such as clinical examination, signs/symptoms and medical history, and biological information such as biomarkers (CRP and WBC) and pathogen-specific PoC tests, and oximetry will be developed. The e-algorithm will serve to guide the diagnostic and management of febrile infections in children.

In the 3 arms, the case report forms will be digitalized. A final follow-up visit (day 7) will be scheduled for all participants. Patients will be asked to come back to the health facilities before the scheduled visit if the symptoms persist or in case of health condition worsening.

**Discussion:**

If successful, this study could contribute to improve the management of febrile diseases and reduce inappropriate use of antimicrobials.

**Trial registration:**

The trial is registered at ClinicalTrial.gov, NCT05285657. Enrolment started on 4 March 2022 with long-term outcome being assessed completely by 2023.

## Administrative information

Note: the numbers in curly brackets in this protocol refer to SPIRIT checklist item numbers. The order of the items has been modified to group similar items (see http://www.equator-network.org/reporting-guidelines/spirit-2013-statement-defining-standard-protocol-items-for-clinical-trials/).

**Table:** 

Title {1}	Development and Evaluation of an Electronic Algorithm Using a Combination of a Two-step Malaria RDT, and Other Rapid Diagnostic Tools for the Management of Febrile Illness in Children Under 5 Attending Outpatient Facilities in Burkina Faso (e-MANIC).
Trial registration {2a and 2b}.	NCT05285657 14/03/2022 Clinicaltrials.gov.
Protocol version {3}	01/10/2021, Version 02.
Funding {4}	European & Developing Countries Clinical Trials Partnership (EDCTP) through the EDCTP career Development Fellowship TMA2019CDF-2697.
Author details {5a}	^1^Institut de Recherche en Sciences de la Santé – Clinical Research Unit of Nanoro (IRSS-CRUN), Nanoro, Burkina Faso ^2^Department of Pediatrics – CHU Yalgado Ouedraogo, Ouagadougou, Burkina Faso ^3^Health District of Nanoro, Ministry of Health, Nanoro, Burkina Faso ^4^National Malaria Control Program, Ministry of Health, Ouagadougou, Burkina Faso ^5^National Agency for Primary Healthcare, Ministry of Health, Ouagadougou, Burkina Faso ^6^Amsterdam University Medical Centers, Academic Medical Centre at the University of Amsterdam, Department of Medical Microbiology and Infection Prevention, Laboratory for Experimental Parasitology, Amsterdam Institute for Infection and Immunity, Meibergdreef 9, 1105 AZ Amsterdam, The Netherlands.
Name and contact information for the trial sponsor {5b}	Institut de Recherche en Sciences de la Santé – Clinical Research Unit of Nanoro (IRSS-CRUN), 11 BP 218 Ouaga CMS 11; T.: +226 25 40 92 12/25 44 62 49; Fax: +226 +226 25 40 92 12.
Role of sponsor {5c}	The sponsor is responsible for the study protocol development and ensure that proper arrangements are in place to initiate, manage and report the study.

## Introduction

### Background and rationale {6a}

Fever is a very general clinical symptom in pediatric patients and a common cause to take a child to the doctor or health worker [1]. Febrile illnesses have various etiologies (parasites, bacteria and viruses) [2] and are the common causes of morbidity and mortality in low-and middle-income countries (LMICs) [2, 3]. In Burkina Faso, children are managed presumptively according to the Burkinabe national guidelines based on WHO guidelines for the Integrated Management of Childhood Illness (IMCI), except for malaria. Other causes of fever are managed without knowing the actual etiology [4]. This practice lead to the inappropriate prescription of antimicrobial.

During the last 10 years, interest for point-of-care (PoC) tests that can be implemented in LMIC has grown in order to achieve the 2030 Sustainable Development Goal 3 (SDG3). The introduction of malaria RDT in the routine health system ranks high among the major and feasible innovations in the management of febrile diseases in endemic areas. Indeed, this introduction has considerably reduced the unnecessary prescription of antimalarials and saved lives in vulnerable groups such as children under 5 years [5]. However, the persistence of HRP2 antigen after successful antimalarial treatments and the low sensitivity of *p*LDH RDT negatively influences the prescription of antimalarials and antibiotics [6–8]. This raises questions about the appropriateness of one-step malaria RDT only, to correctly manage febrile diseases in primary health facilities. Importantly, *Pf*HRP2-based RDT is useful for the active detection of new malaria infection and *p*LDH for the monitoring of parasite clearance [6, 9–14].

In a recent study conducted at CRUN, it has been reported that the implementation of an algorithm combining two-step malaria RDT detecting *Pf*HRP2/*p*LDH and information on previous antimalarial treatment can improve the diagnosis of malaria compared to the use of single malaria RDT only [15]. Although this two-step malaria RDT was found to improve malaria diagnostic, the lack of PoC tests for non-malaria infections in primary health facilities such as biomarkers (C-reactive protein and white blood cell count) and bacterial RDTs to guide clinical diagnostic may lead to the inadequate management of the patients and unnecessary prescription of antibiotics. This practice is one of the leading causes of antimicrobial resistance. Capitalizing on the successful management of malaria and human immunodeficiency virus (HIV) due to the deployment of RDTs to these diseases [16, 17] and the introduction of diagnostic kits for the rapid diagnosis of other infections will create an opportunity to improve the management of febrile diseases in areas without laboratory facilities. In order to promote access to health care of quality for the population and reduce inappropriate use of antimicrobials, Burkina Faso has adopted a policy of universal health coverage (UHC). Correct, timely, and cheaper diagnosis of infectious diseases remains a cornerstone for appropriate therapeutic interventions within reasonable time to avoid fatalities due to late and/or wrong prescriptions of antimicrobials [18–20]. Before the algorithm combining two-step malaria RDT and innovative PoC tests for bacterial infection can be recommended in routine care in rural settings, their real value for the management of febrile diseases should be evaluated medically, biologically, socially, and economically in the field.

The objective of this study is to assess the appropriateness and the added value of an algorithm including two-step malaria RDT detecting *Pf*HRP2/*p*LDH as well as biomarkers and specific antigen PoC tests to guide bacterial infection, for the management of febrile illnesses in primary health facilities. This proposed algorithm could enhance the UHC and at the same time improve malaria diagnosis and reduce unnecessary antimicrobials prescriptions. The results of this study will serve as a potential path of policy change that aims to support the Integrated Management for Childhood Illness (IMCI) with an evidence-based diagnosis for better impact. By relying on IMCI and guideline of diagnostic and existing treatment in Burkina Faso, the policy implementation of this joint intervention will be scalable at country and regional levels.

### Objectives {7}

This study aims to evaluate the value of the diagnostic performance of the proposed algorithm combining two-step malaria RDT detecting *Pf*HRP2/*p*LDH and PoC tests for the diagnosis of malaria and bacterial infections respectively, in the management of febrile illnesses in children from 6 to 59 months in a process of UHC.

The PoC tests expected to be performed in this study are malaria RDTs (*Pf*HRP2/*p*LDH), C-reactive protein RDT, white blood cell (WBC) count, urine dipstick, pulse oximeter, group A *Streptococcus* RDT, RDT for *Salmonella*, and *Shigella* for rapid detection in stool.

### Trial design {8}

This study will be an open-label, superiority, three-arm individual randomized controlled diagnostic trial to evaluate a new algorithm including two-step malaria RDT and PoC tests for bacterial infections: (1) control arm, (2) RDT decisional algorithm (RDT-DA) arm, and (3) e-algorithm arm.

In the 3 arms, healthcare workers will be trained to perform properly the clinical examination.

- In the control arm, the diagnostic and management will be based on IMCI and guidelines of diagnostic and treatment (GDT). The management of the participant will be only supported by a one-step malaria RDT, rolled-out by the MoH for malaria management in primary health facilities. The diagnosis and prescription will be left to the discretion of the healthcare workers, based on the Integrated Management of Childhood Illnesses (IMCI) and GDT.
- In the RDT-DA arm, the presumptive diagnostic will base on IMCI and guidelines of diagnostic and treatment (GDT). This presumptive diagnostic will be supported by biomarkers PoC diagnostic tools (CRP, WBC and urine dipstick), pathogen-specific antigen RDT (two step malaria RDT and bacteria), and pulse oximetry to determine if a prescription of antibiotic is needed. However, in this arm, the diagnosis and prescription will be left to the discretion of the healthcare workers, based on clinical examination and PoC test results.
- In the e-Algorithm arm, the clinical examination will be supported by the two-step malaria RDT and the PoC tests for bacterial infections to determine if a prescription of antibiotic is needed. However, in this arm, the clinical evidence and the results of malaria two-step RDT and the PoC tests for bacterial infections will be digitalized to guide diagnostic and propose treatment. After the properly completion of the e-Algorithm, the final diagnosis will be done by the algorithm as well as the prescriptions (antibiotics and antimalarials mainly).

In the decisional algorithm arms (RDT-DA) and e-Algorithm arms, the PoC tests will be done by trained nurses.

In the 3 arms, the study CRFs (case report forms) of data collection will be digitalized on tablets for an electronic data collection.

The implementation of the algorithm will be based on the implementation of PoC tests to support clinical diagnostic and guide treatments. Study participants (children) will be identified during their visit at the study health facilities. Children attending the health facilities and fulfilling the inclusion criteria will be included in the study only if their parents/legal guardian signs an informed consent form. Upon the inclusion, children will be randomized to be allocated either in the control, RDT-DA algorithm, or e-algorithm arms. After the initiation of the treatment, the health outcome will be assessed at day 7. Those who will not return to the health facilities for day 7 visit will be visited at home by a field worker to collect the information. Patients will be asked to come back to the health facilities before day 7 if their health condition is not improving or is getting worse. Study will be ended for the child if at day 7 visit, clinical signs, and symptoms are no longer presents; otherwise, the participant will be referred to the study clinician or pediatrician for better care.

## Methods: participants, interventions, and outcomes

### Study setting {9}

The study will take place at the Clinical Research Unit of Nanoro (CRUN) field station of Siglé in Burkina Faso. The peripheral Health facilities of Siglé and Pella have been selected to be part of this study based on the number of consultation of this age group for fever or history of fever at these health facilities in order to be sure to reach the sample size on time (1 year recruitment). A monthly target enrolment plan has been developed based on the monthly attendance at each health facility in 2018 (Table 1).

**Table 1:** Summary of monthly consultations and recruitments

Month	Total Pella (consultation)	Proportion Pella (consultation)	Total Siglé (consultation)	Proportion Siglé (consultation)	Ratio P:T	Total	Proportion total	Target enrolment total	Target enrolment Siglé	Target enrolment Pella	Cumulative Siglé	Cumulative Pella	Cumulative total	Cumulative total
December	531	10	195	7	3.7	726	9	102	27	75	27	75	102	9%
January	245	5	185	6	2.3	430	5	61	26	35	54	109	163	14%
February	253	5	232	8	2.1	485	6	68	33	36	86	145	231	20%
March	121	2	120	4	2.0	241	3	34	17	17	103	162	265	23%
April	73	1	146	5	1.5	219	3	31	21	10	124	172	296	25%
May	166	3	138	5	2.2	304	4	43	19	23	143	196	339	29%
June	125	2	92	3	2.4	217	3	31	13	18	156	213	370	31%
July	306	6	184	6	2.7	490	6	69	26	43	182	257	439	37%
August	901	17	442	15	3.0	1343	16	189	62	127	245	384	628	53%
September	1003	19	439	15	3.3	1442	17	203	62	141	306	525	832	71%
October	903	17	347	12	3.6	1250	15	176	49	127	355	652	1008	86%
November	761	14	432	15	2.8	1193	14	168	61	107	416	760	1176	100%
**Total**	5388	100	2952	100	2.8	8340	100	1176	416	760				

In this setting, primary health care is provided by nurses and only severe cases are referred at the Nanoro referral hospital for better care. Clinical signs and symptoms remain the only diagnostic tools available for the diagnostic of infectious diseases, except for malaria for which a one-step RDT detecting HRP2 have been introduced in routine practices in 2010. According to an etiological study conducted by the CRUN, almost half of children under 5 years of age living in this area are considered not to be infected by malaria. This raises the dilemma for health care workers to correctly manage febrile episodes in children under 5 years when malaria is rolled-out. This situation becomes exacerbated when the RDTs use is below the expected diagnostic accuracy [6–8]. Unfortunately, bacterial infections are treated presumptively based on guidelines established almost 10 ago [21].

### Eligibility criteria {10}

The study will include patients who fulfill the following inclusion criteria:

1. Children from 6 to 59 months of age;
2. Acute fever (axillary temperature≥ 37.5°) or history of fever within the past 7 days;
3. Available to return for the follow-up visit at the health facility on day 7 (± 2).
4. Written informed consent obtained from parents/legal guardian.

Patients will be excluded from the study if they meet any of the following conditions:

1. Children less than 6 months or over 59 months;
2. Presence of signs and symptoms of severe infections;
3. Children with chronic febrile infections (over 7 days).

### Who will take informed consent? {26a}

Before the implementation of the study, community through their leaders will be informed about the purpose of the study. All discussions will be conducted in the native language of the patients by a qualified person identified by the Investigator. Written information and consent forms will be provided to the parent/legal guardian. After the discussion, the patients/legal guardian of the participant will be asked to confirm their willingness to let his/her child participate in the study by signing (or thumb-printing whenever they are illiterate) the consent form. The informed consent form will describe the purpose of the study, the procedures to be followed, the risks and benefits of participation, etc. If a parent/legal guardian is unable to read or write, a signature from an impartial witness participating to the informed consent discussion will be obtained. Parents/legal guardians will be informed that participation in the study is completely voluntary and that they may withdraw his/her child from the study at any time without any negative consequences.

### Additional consent provisions for collection and use of participant data and biological specimens {26b}

In the inform consent procedure, study participants (and/or parents/guardians) will be informed about future investigations on the collected blood samples. They will be explained that future investigations will only focus on the understanding of the performance and quality of diagnostic procedures. Genetic testing using materials from the human study subject will not be performed.

### Interventions

#### Explanation for the choice of comparators {6b}

The routine practice in place at the recruitment clinic will serve as a comparator of the proposed algorithm (e-Algorithm and decisional algorithm). However, a follow-up visit is planned for participants of the three arms at the health facility.

### Intervention description {11a}

#### Development of the e-Algorithm

Given the lack of clinicians in remote area for the management of infectious diseases, guidelines such as the “Integrated Management of Childhood Illnesses (IMCI)” and the “guideline of diagnostic and treatment (GDT)” have been proposed to help nurses for the management of febrile infections in primary healthcare settings based on clinical signs and symptoms, except malaria. The fear to overlook potential and treatable infection due to the limitations of malaria RDTs to correctly diagnose malaria infection and the absence of practical tool to screen other causes of fever lead health care workers to prescribe antimicrobial to most of febrile children without malaria or bacterial infections. In this study, we propose to develop an e-Algorithm using diagnostic tools (PoC tests) to support presumptive diagnostic and guide final diagnostic and treatment.

The e-algorithm proposed in this study is an artificial intelligence (AI) platform integrating multiple layers of clinical information for the health care of acute febrile illnesses. This artificial intelligence will integrate the following: (1) clinical parameters as described in IMCI for febrile illnesses; (2) clinical signs/symptoms specific to each infection; (3) medical history; and (4) PoC test results such as biomarkers (CRP, WBC, and urine dipstick) and pathogen-specific PoC tests and oximetry. The AI should be able to identify meaningful relationships in raw data based on clinical and biological evidence and will have the potential to be applied in primary health facilities. Data will be captured directly into the algorithm developed. The data capture will guide the diagnostic by requesting other clinical information and laboratory tests needed to guide diagnostics and treatments. The proposed algorithm at the end of clinical consultation should guide diagnostic and prescription.

This intervention (e-algorithm and RDT-DA) will be developed by a panel of researchers working on pediatric care such as pediatricians (professional and academic), clinicians, nurses, biologists, and computer program developers. The IRSS-CRUN has the required expertise in the development of artificial intelligence and could put this expertise in the development of the proposed artificial intelligence for health care at primary health facilities.

The validation of the algorithm will be done at the first workshop (M6) with stakeholders of the Ministry of Health (MoH) before the enrolment of the first participant.

#### Study tests

The following diagnostic tests will be used:

- Two-step malaria RDT detecting *Pf*HRP2/*p*LDH (SD Ag Bioline Malaria Ag *P.f*/*Pan*: Standard Diagnostics, Hagal-Dong, Korea);
- C-reactive protein (CRP) test (SD Biosensor Standard F 100 quantitative CRP);
- HemoCue® WBC DIFF System;
- Urine dipstick (UroColor, Standard Diagnostic Inc., Korea);
- Pulse oximeter (BESCO Fingertip Pulse oximeter, BES 500D);
- Pharyngitis: Streptococcus A throat test (SD BIOLINE Strep A);
- *Salmonella* and *Shigella* tests for diarrheal/dysentery (CERTEST Biotech, *Salmonella* detection kit and *Shigella* detection kit).

#### Interpretation of the two-step malaria RDT detecting PfHRP2/pLDH for the diagnostic of malaria and other point-of-care (PoC) tests

- The two-step malaria RDT detecting *Pf*HRP2/*p*LDH

**Fig. 1:**
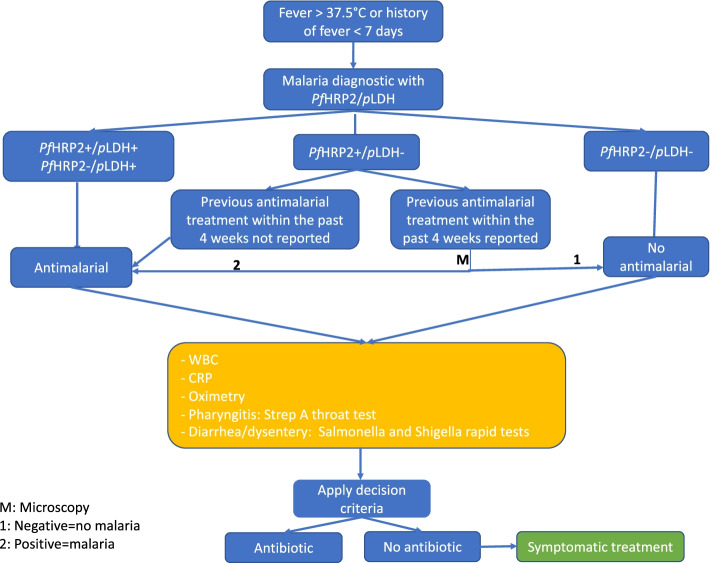
Proposed algorithm. ATB, antibiotic; CRP, C-reactive protein; WBC, white blood cells

The management of fever episode will be based on clinical decision algorithm proposed below (see Fig. 1). Each febrile child (fever or history of fever) attending the health facilities and meeting the eligibility criteria will be invited to participate in the study. A two-step malaria RDT will be tested for all the participants of the intervention arms. The interpretation of the two-step malaria RDT will be done as follow:

- *Pf*HRP2(+)/*p*LDH(+): falciparum malaria or co-infection with non-falciparum malaria;
- *Pf*HRP2(−)/*p*LDH(+): non-falciparum malaria or falciparum malaria with deletion of *hrp*2;
- *Pf*HRP2(−)/*p*LDH(−): negative result
- *Pf*HRP2(+)/*p*LDH(−): inconclusive result and information on previous antimalarial treatment is needed to differentiate:
  - ◦ If previous antimalarial treatment *is* reported within the past 4 weeks (< 28 days), the malaria diagnosis will be reported as *negative*. Nonetheless, the antimalarial treatment decision will be based on malaria microscopy;
  - ◦ If previous antimalarial treatment *is not* reported within the past 4 weeks (< 28 days), the malaria diagnosis will be reported as *positive*.
- Other PoC tests for bacterial infections

The CRP and WBC count will be tested to diagnose bacterial or viral infection in all patients. The oxygen saturation will be measured with an oximeter to diagnose pulmonary infection by combining this result with clinical signs and symptoms. The implementation of *Streptococcus* A throat test and *Salmonella* and *Shigella* tests for diarrheal/dysentery will be based on clinical signs and symptoms. The urine dipstick test will be based on the presence of signs of urinary tract infections.

In the RDT-DA arm, the other PoC tests will be performed at the discretion of the healthcare workers and on the basis of clinical signs and symptoms reported during the physical examination. In the e-Algorithm arm, clinical signs and symptoms and other clinical information necessary to guide the diagnosis, and the results of the two-step malaria RDT and PoC tests for bacterial infections will be recorded directly on the AI platform to guide the final diagnostic and propose treatment.

Any suspected case of COVID-19 during the study implementation will be referred to the local COVID-19 riposte team based at the district hospital of Nanoro.

The treatment will depend on the clinical evidence and the results of the tests. In the RDT-DA algorithm arm, the prescription will be done at the discretion of the health care workers. However, in the e-Algorithm arm, the complete clinical examination and the outcomes of RDTs (malaria and bacterial infections) will be recorded on the AI platform. Diagnosis and prescription will be done by the algorithm. Tables 2, 3, and 4 show the decision of treatment according to clinical signs and symptoms.

**Table 2:** Presence of respiratory signs and symptoms

	***Strep A test***	***If at least 1 positive***	***If the both negative***
Oxygen saturation	90–95%	ATB	(1) or (2)
	> 95%	ATB	(2) Consider CRP and WBC count

(1) Consider antibiotic treatment if patient fill the criteria for pneumonia according to WHO or basing on clinical consideration

(2) Consider CRP and WBC count

*ATB* antibiotic, *CRP* C-reactive protein, *WBC* white blood cells

**Table 3:** Presence of gastroenteritis signs and symptoms

***Salmonella and/or Shigella***	***If positive***	***If negative***
Action	ATB	Consider CRP and WBC

*ATB* antibiotic, *CRP* C-reactive protein, *WBC* white blood cells

**Table 4:**
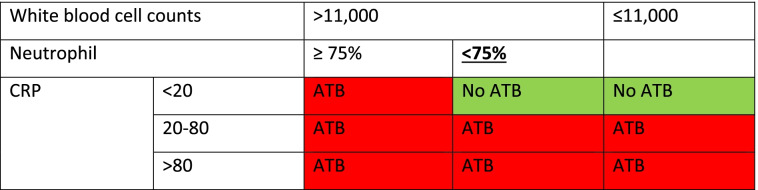
WBC and CRP guided decision

### Enrolment

#### Day 0: Screening visit/ sample collection

1. Informed consent: After an in-depth discussion with the study physician or a member of the study team, the parent/legal guardian of the potential participant will be asked to document his consent by signing an informed consent form. The signed informed consent form (or thumb-print whenever the patient or the parents/guardians are illiterate) must be obtained before any data or sample collection for the study is done. Patients/guardians who will not consent to let his/her child to be part of the study will receive the standard of care as recommended by the Ministry of Health of Burkina Faso.
2. Randomization of study participants: Study participants will be randomized to routine, RDT-DA or e-Algorithm arm at a ratio of 1:1:1 using varying block size of 20, 30, or 40 subjects in random order. Randomization list will be prepared by the data management and (bio) statistic department of CRUN.
  Randomization of study participants into the three arms will be performed by a trained study staff, after checking the inclusion and exclusion criteria.
3. Demographic data and medical history: Demographic data and a general medical history of past and present illnesses, including the intake of any medication in the past month, will be recorded to determine the characteristics of the study participants.
4. Control and intervention arms

- Control arm:

In the control arm, participants will follow the routine standard of care for the management of febrile diseases according to the national guidelines. In this arm, HRP2 based malaria RDT will be the only diagnostic tool available for the management of the fever episode. The clinical assessment (history of presenting complaints and physical examination) will be performed by health facilities staff (nurses). Except malaria for which an RDT is available, the other prescriptions in this arm will be based on clinical signs and symptoms as defined in IMCI.

- RDT-DA arm

The clinical assessment of the participant (history of presenting complaints and physical examination) will be performed by the study nurses. In this arm, participants will benefit the two-step malaria RDT detecting *Pf*HRP2/*p*LDH for the diagnosis of malaria infection and PoC tests for the diagnostic of bacterial infections. Samples tested will be sent to the microbiology laboratory of CRUN for culture.

However, in this arm, the diagnosis and prescription will be left to the discretion of the healthcare workers, based on clinical examination and PoC test results and basing Fig. 1 and Tables 2, 3, and 4.

- e-Algorithm arm

The clinical assessment of the participant (history of presenting complaints and physical examination) will be performed by the study nurses. In this arm, participants will benefit the two-step malaria RDT detecting *Pf*HRP2/*p*LDH for the diagnosis of malaria infection and PoC tests for the diagnostic of bacterial infections. Samples tested will be sent to the microbiology laboratory of CRUN for culture.

However, in this arm, the clinical evidence and the results of malaria two-step RDT and the PoC tests for bacterial infections will be digitalized to guide diagnostic and propose treatment. After the properly completion of the e-Algorithm, the final diagnosis will be done by the algorithm as well the prescriptions (antibiotics and antimalarials mainly).

#### Day 7: Follow up

Study participants will be actively followed-up by the study team (control arm, RDT-DA arm and e-Algorithm arm) on day 7 to assess their health status as well as find out if they procured other treatment different to those prescribed at the health facilities. If the participants do not come back for the control at day 7, a home visit will be performed by study staff to collect the information. For that, an additional 2 days will be given to collect this information. The caregivers will also be interviewed by social scientists the day of their follow-up (day 7) either at the health facility or at a place indicated by the caregiver if he feels this place will be better for him to freely express himself. In both cases, the place should meet the requirements for confidentiality and patients’ protection.

#### Unscheduled visits before day 7

All patients will be asked to visit the health facility at any time if the child health status is not improving or in case or worsening. This will be documented as unscheduled visit and reported in the CRF.

### Criteria for discontinuing or modifying allocated interventions {11b}

It is hypothesized that the proposed algorithm will reduce inappropriate prescription of antibiotics and antimalarials in intervention arms. However, an efficient follow-up system will be putted in place to ensure the reporting and management of adverse events requiring medical care on time. In case of frequent occurrence of related serious adverse events, the intervention will be interrupted.

### Strategies to improve adherence to interventions {11c}

The proposed tests are available on the market but are not part of the routine health system at primary health facilities. Prior to the implementation of the intervention, an explorative research will be conducted in the study area to investigate the factors that may impact on the acceptability and feasibility of implementing the intervention (introduction of new POC tests) in the routine health system, mainly among health care workers and parents/guardians. This explorative research will be conducted by a trained social science team. The outcomes of this explorative research will serve to train study staff to adopt good attitude with study participants and encourage them to adhere to study conditions during the follow-up visit.

Each participant, regardless of his randomized arm, will be follow-up at the day 7 (± 2) at the health facility to assess the health status of the child and the adherence of the parent/guardian to the prescription. The adhesion to the prescription will be assessed through a qualitative in-depth interview of parent/guardian and pill count (parents/guardians will be asked to bring back packaging and remaining medicine on day 7 visit), including behavior of parent/guardian who will not receive prescription of medication to find out if they purchased any antibiotic or any other treatment, the place of purchasing, and the reasons.

Participants who do not return at the health facilities at day 7 will be followed-up at home or by telephone to assess their wellbeing and the need to return at the health facilities.

### Relevant concomitant care permitted or prohibited during the trial {11d}

Parents/guardians will be asked to return at the recruitment site with the participants if the symptoms persist and/or health conditions worsening. However, participation in the study is voluntary and parents are free to judge the need for concomitant care for their children. Nevertheless, parents/guardians will be asked to report any concomitant care to the study staff. No routine interventions are prohibited during the trial.

### Provisions for post-trial care {30}

Given the fact that this is a diagnostic trial and not an interventional clinical trial testing a new drug/device, such visits are not needed.

### Outcomes {12}

Primary study outcomes will be:

(i) The rate of febrile cases with favorable outcomes at day 7 in each arm;
(ii) The proportion of antimalarial and antibiotic prescriptions for acute febrile at the health facilities in each arm at day 0.

Secondary outcomes include:

(i) The adherence of healthcare workers to the algorithm at day 0;
(ii) The adherence of parents/guardian to treatment. This will be assessed at day 7 visit;
(iii) Accuracy of the algorithm for the diagnosis of malaria and others PoC tests to guide bacterial infections (biomarkers and pathogen-specific PoC tests). This will be assessed at day 7;
(iv) The safety of the algorithm for the management of febrile diseases. This will be assessed at day 7 visit.

### Participant timeline {13}

The algorithm implementation will be based on clinical evidence that the healthcare workers will record and the test results. Study participants (children) will be identified during their visit at the health facilities. Children attending the health facilities and fulfilling the inclusion criteria will be included in this study only if their parents/legal guardian signs the informed consent forms. After signature, children will be randomized to be allocated to either in control, RDT-DA algorithm or e-algorithm arms. After the initiation of the treatment, the health outcome will be assessed at day 7. Parents will be asked to come back to the health facilities with his/her child before day 7 visit if his/her health condition is not improving or is getting worse. Study will be ended for a child if at day 7 visit clinical signs and symptoms are no longer presents; otherwise, he will be referred to the study clinician or pediatrician for better care (Table 5).

**Table 5:**
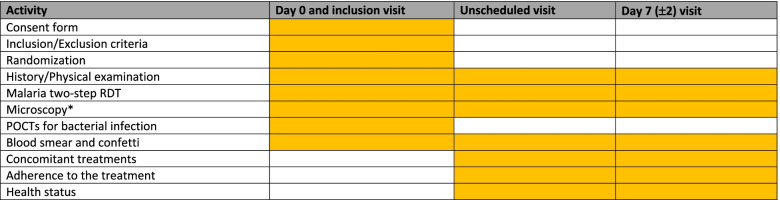
Study flow chart

### Sample size {14}

Sample size is calculated based on data collected in the Siglé and Pella health facilities’ consultation books. From these data collected in 2018 (January to December), the proportion of febrile children under 5 years presenting at the health facilities with febrile illnesses and who were prescribed antibiotic was 62.63%. The implementation of the algorithm is expected to reduce this proportion to at least 20%. In this study, the sample size is computed to a Bonferroni correction of alpha which is performed for multiple comparisons; therefore, an alpha level of 0.017 is chosen. Type II error was controlled at 20%, i.e., (*β* = 0.20 or 80% power). Based on these parameters, a sample size of 356 is required per arm. Hence, an overall sample size for the study is estimated at 1068 (3 × 356). Further assuming that 10% of the individuals will be lost to follow-up, an adjusted sample size of 392 children per arm is required. The final estimated sample size for this study is thus 1176 children.

### Recruitment {15}

Two health facilities have been selected to be part of this study based on the consultation of this age group at these health facilities to be sure to reach the sample size on time: Siglé and Pella. A monthly target enrolment plan which covers the whole year recruitment will be developed based on the monthly attendance at each health facility before the enrolment of the first participant. This target enrolment plan will be followed by the study team to be sure that the sample size will be reached on time and make some adjustment if needed (Table 1).

### Assignment of interventions: allocation

#### Sequence generation {16a}

This will be an open-label randomized controlled trial with three arms, enrolling febrile children from 6 to 59 months. A computer-generated randomization list will be prepared and generated by the data management department of CRUN. Only the central data management team will have access to the randomization list.

#### Concealment mechanism {16b}

Each randomization number and corresponding group (e-Algorithm, decisional algorithm and routine) will be sealed in an opaque envelope which will only be opened by study staff after enrolment of the participant.

#### Implementation {16c}

The central data management team of IRSS-DRCO will generate the allocation sequence. Participants will be enrolled by nurses who will assign participants to interventions (e-Algorithm, decisional algorithm arms) after confirmation of all eligibility criteria.

### Assignment of interventions: blinding

#### Who will be blinded {17a}

This is an open-label trial. Therefore, this is not applicable to this study. Nevertheless, during data analysis, data analysts will not have access to the identity of the participants or any information that can link participant to the data. The finding will be reported in summary form.

#### Procedure for unblinding if needed {17b}

This is an open-label trial. Therefore, this is not applicable to this study.

### Data collection and management

#### Plans for assessment and collection of outcomes {18a}

A standard case report form (CRF) will be used to collect details on medical history and clinical examination, as well as the medical prescription in each group. The CRF will be digitalized for each arm.

For this study, an algorithm with functionalities easy to be used by nurses working at primary health facilities will be developed and installed on tablets for testing. All the clinical information needed to make a clinical decision based on IMCI and GDT will be pre-recorded and digitalized for the study use.

In control arm, nurses will systematically collect and enter all information related to fever during physical examination in the e-CRF. This physical examination will be supported by the malaria RDT, which is the only diagnostic tool available at the primary health facilities.

In the RDT-DA arm, nurses will systematically collect and enter all information related to fever during physical examination in the e-CRF. This physical examination will be supported by the POC tests, left to the discretion of the nurses. The final diagnostic and the prescription will be also left at the discretion of the nurses.

In the e-Algorithm arm, nurses will systematically collect and enter all information related to fever during physical examination in the e-CRF. This physical examination will be supported by the POC tests. A program will be set up by combining clinical signs and symptoms as well as the results of the PoC tests and will be downloaded on tablets to guide the diagnosis and the prescription.

All enrolled participants will be followed up on day 7 to assess their health status as well as find out if they complied with the prescription.

If fever persists, the participant will be referred to see a pediatrician at CRUN for better care. All treatment provided will be collected as well as the outcomes of the diseases. Parents/legal guardian will be asked to report to the clinical study staff at the health facilities, all concomitant treatment administered to the participant during the follow-up.

#### Plans to promote participant retention and complete follow-up {18b}

This study is planned for 7 days follow-up and loss to follow-up could be an issue even if the risk is low. To prevent this and based on our previous experience, an anticipated 10% corrective measure has been allocated to this risk in the sample size calculation to prevent any loss of follow-up. An explorative research has been conducted to assess the factors that could facilitate the acceptability of the proposed algorithm at community level. However, study staff will insist on communication with community/parents/guardians before the enrolment in the study in order to improve knowledge and awareness of study tests as well as the purpose of the study.

#### Data management {19}

Appropriate individual case record forms (CRFs) will be developed for data collection. In control and RDT-DA algorithm arms, CRF will be digitalized for the data collection only. However, in e-Algorithm arm, the algorithm including two-step malaria RDT detecting *Pf*HRP2/*p*LDH and PoC tests for bacterial infection will be digitalized to assist the healthcare workers in the diagnostic and treatment process and give the final diagnostic and the treatment proposed. For this purpose, an electronic CRF (e-CRF) will be developed. Adequate procedures will be put in place to ensure accuracy, reliability, and consistency of electronic records in e-Algorithm arm. The data management system will include audit systems to independently record the date and time of operator entries and actions that create, modify, or delete electronic records. All the data will be backed up and archived periodically. To avoid any entry of inconsistency data, a data entry control has been integrated in the digitalization of the CRFs. Nevertheless, a system of management of queries has been set-up to generate systematically queries and addressed to the study clinician for correction if any.

#### Confidentiality {27}

All study documents and supports are provided in confidence to the investigators and his/her appointed staff. None of this material may be disclosed to any party not directly involved in the study without written permission from the sponsor.

#### Plans for collection, laboratory evaluation, and storage of biological specimens for genetic or molecular analysis in this trial/future use {33}

Several biological samples will be collected for the management of participants depending on the randomization arms. In addition, biological samples will be collected for malaria microscopy and molecular analysis with PCR, and microbiology tests. These works will be restricted to the assessment of the presence of *Plasmodium falciparum* and other bacterial species. No human genetic material will be analyzed. The samples collected could be used for other analyses in order to improve the diagnostic of febrile diseases and specific informed consent will be obtained from study participants for this.

### Statistical methods

#### Statistical methods for primary and secondary outcomes {20a}

An analysis plan of the data has been developed and validated by the participants of the first workshop of the project, prior to the recruitment of the first study participants. Briefly, statistical analysis will be presented by treatment group. Categorical variables will be summarized as proportion, and chi-square test or Fisher’s exact test will be performed. For continuous variables, they will be described by mean or median and compared by using Student’s *t*-test. The proportion of patients that meet the criteria of the primary outcome (proportion of recovered case and antimicrobial) will be presented by arm. This analysis plan could be revised according to events and in case of amendment of the protocol. The statistical analysis will be done with an appropriate data analysis software (STATA 14.1. and R).

#### Interim analyses {21b}

Interim analyses are not planned.

#### Methods for additional analyses (e.g., subgroup analyses) {20b}

Next to the assessment of the proposed algorithm combining two-step malaria RDT detecting *Pf*HRP2/*p*LDH and PoC tests for bacterial infections, a social science investigation and an economic evaluation of the intervention will be conducted to identify the bottlenecks of the implementation of the point-of-care tests at primary health facilities level and the cost-effectiveness analysis of the intervention.

#### Design of social science investigation

The social sciences investigation will be focused on the adherence of healthcare workers to the algorithm (RDT-DA and e-Algorithm) as well as their prescription and patients/guardians’ adherence to the treatment prescription. The social science investigation will occur in two phases.

#### Phase 1: Exploration research

This research will occur immediately months 4 and 5) after the approval of the protocol by the ethics committee. The aim of this investigation will be to identify factors that may impact on the acceptability and feasibility of implementing the intervention (introduction of new POC tests) in the routine system, mainly among health care workers and parents/guardians.

*Study population*: To achieve the goal of this first step, three different populations will be considered for the qualitative data collection: healthcare workers, parents/guardians, and community members who are the main decision makers in the household.

*Sampling and sample size estimation*: Participants will be purposively selected and recruited from 3 selected populations based on some characteristics:

- The healthcare workers assigned by the MoH at the health center with at least two (2) years of experience. A maximum of 5 healthcare workers will be interviewed per site. This sample has been determined based on the number of nurses appointed at the health facilities of Siglé, Pella, and Bologo (maximum 5 nurses by health facility).
- The parents/guardians interviewed will be the ones attending the health facility with febrile children less than 5 years. A sample of 5 staff will be interviewed per site, which number can be reconsidered according to saturation of content principle.
- Two group discussions will be done with 16 community members, a group of 8 males and 8 females from the area where the health care center is located and who are the main decision makers in the household.

*Data collection tools*: Data (in-depth interview and discussions) will be audio recorded under the consent of the participants.

*Data analysis*: Data collected will be transcribed manually. NVivo software will be used for the management and the coding of data. During the analysis, key topics and concepts generated will be categorized.

*Outcome*:

- Primary outcome: Have a general overview of what can be an obstacle or strength for the study implementation.
- Secondary outcome: Create a good climate for study implementation.

#### Phase 2: Evaluation research

This research will occur during the study implementation. The aim of this investigation will be to analyze the adherence of health care workers and the community to the new algorithm. The participants will be interviewed on day 7 when they will come for the follow-up. This will help minimize forgetfulness.

*Study population*: The study population will be the healthcare workers who implemented the study in the different sites and the parents/guardians.

*Sampling and sample size estimation*: The healthcare workers who will participate in the study are those who implemented the study on the site (six nurses are expected for the two sites) and the parents/legal guardians of the study participants attending the recruitment sites (participants will be interviewed until the saturation of content is reached).

*Data collection tools*: Data (in-depth interview) will be audio-recorded under the consent of the participants.

*Data analysis*: Data collected will be transcribed manually. NVivo software will be used for the management and the coding of data. During the analysis, key topics and concepts generated will be categorized.

*Outcome of the investigation*:

- Primary outcome: Analyze the adherence of parents/guardians and health care workers to the algorithm.

#### Conception of health economic survey

The objective of the health economics component is to estimate the cost-effectiveness ratio of the proposed algorithm compared to the detection practices in place in the health facilities of the Nanoro health district.

#### Specific objectives

- Estimate the cost of the proposed algorithm in the health facilities of the Nanoro health district;
- Estimate the costs of routine practices used in health facilities in the Nanoro health district;
- Calculate the incremental cost-effectiveness ratio;
- Determine the most cost-effective algorithm.

#### Methodology

*Type of survey*: A prospective study will be implemented to evaluate the cost effectiveness of the proposed algorithm in comparison with the routine algorithm in health facilities.

*Study sites*: The study sites are those of the e-MANIC study, namely the health centers of Siglé and Pella.

*Study perspective*: In Burkina Faso, children under 5 years of age are part of the UHC policy. So, all costs related to their healthcare are free of charge for parents/guardians. For that, we assumed that all costs were borne by the health facility, even those borne by the government such as salaries of health staff in each group (control and intervention). In short, our study perspective will be that of the care provider.

*Cost estimate*: To compare a medical intervention with another with the same objective, the most appropriate type of economic evaluation is a cost-effectiveness study. The two algorithms have the same efficiency indicator, namely the rate of cure and prescription of antimicrobials (antimalarials and antibiotics).

The approach taken for cost estimation is that of Larson et al. [22] which was used in a syphilis detection project in Zambia.

This approach proceeded by the inclusion of six parameters which are (1) the cost of the material per test, (2) staff time per test, (3) salary costs per test, (4) daily start-up and quality control costs, (5) cost of equipment, and (6) the average number of tests performed per day. Taking into account the type of algorithm used, we will make a selection of the parameters to include. Already, the costs of equipment such as scales, stethoscope, measuring tape, table, and chair were excluded because their costs are amortized. Additionally, building costs such as examination room, waiting room were also excluded for the same reason. The costs of electricity and the time-motion method will also be used to estimate the time spent to implement the diagnostic algorithms.

*Sample size*: All health facilities in the e-MANIC diagnostic study will be invited to participate in this ancillary study. Health workers from the study and health centers participating in the study will also be invited to participate in the study through interviews to assess the acceptability of the proposed algorithm.

*Collection tools and techniques*: Data collection will consist of observing consultations on the sites and administering a questionnaire based on an interview guide.

The observation technique will be used for the study of time motion. It will make it possible to estimate the time taken for a consultation using the proposed algorithm and in the routine. An observation grid will be developed for this purpose.

The oral interview technique will be used to collect the perceptions of health workers on the acceptability of the algorithm. To this end, individual interviews will be conducted. In addition, a questionnaire will be developed to collect information about the health facility.

*Data analysis*: Quantitative data processing software such as SPSS (Statistical Package for the Social Sciences) and Excel will be used. A data analysis plan will be developed.

The major costs of the different algorithms will be highlighted. The healing rates of each algorithm will be estimated and will serve as an efficiency comparator. The incremental ratio will be calculated according to the following formula:

$$\frac{\mathrm{cure}\ \mathrm{rate}\ \mathrm{of}\ \mathrm{the}\ \mathrm{proposed}\ \mathrm{algorithm}-\mathrm{cure}\ \mathrm{rate}\ \mathrm{of}\ \mathrm{the}\ \mathrm{routine}\ \mathrm{algorithm}}{\mathrm{cost}\ \mathrm{of}\ \mathrm{the}\ \mathrm{proposed}\ \mathrm{algorithm}-\mathrm{costs}\ \mathrm{of}\ \mathrm{the}\ \mathrm{routine}\ \mathrm{algorithm}}$$

The data collected through the individual interviews will be transcribed. A transcription coding grid will be developed. A thematic analysis will follow.

#### Methods in analysis to handle protocol non-adherence and any statistical methods to handle missing data {20c}

The risk of protocol non-adherence is low given the fact that sample collections are based on clinical presentation of the participant and planned only at day 0. Nevertheless, to prevent this, statistical calculation of sample size has been taken this point into account by recruiting an additional 10% of the sample size.

#### Plans to give access to the full protocol, participant level-data and statistical code {31c}

The study has been registered in an accessible clinical trial register and the information contained in that register provides sufficient insight in the full trial design. The final results of the study will be published in peer-reviewed Open Access journals and presented at scientific meetings. None of the trial material may be disclosed to any party not directly involved in the study without written permission from the project sponsor. Presentation and publication of the trial results will be jointly carried out by the project investigators. CRUN will be in charge of sharing relevant results with the respective health authorities. This will be done through ad-hoc meetings, data dissemination workshops, and conferences. Trial data will be accessible for inspection by appropriate health and regulation authorities.

### Oversight and monitoring

#### Composition of the coordinating center and trial steering committee {5d}

Prior to the study start in the field, a trial steering committee will be set-up. This committee will include 8 persons: 2 pediatricians, 2 clinicians, a biostatistician, a representative of the national malaria control programs, a representative of the national agency for primary healthcare, and a representative of the Nanoro health district. The role of this committee is to evaluate the evolution of the trial through regular teleconferences and physical meetings (twice during the implementation of the intervention), advice on the scientific credibility of some actions, and specific issues that may arise during the implementation of the study. Committee decisions will in principle be by consensus. However, in case consensus is not reached, voting will take place and a majority decision will prevail.

#### Composition of the data monitoring committee, its role and reporting structure {21a}

The IRSS-CRUN will contract an independent monitor to ensure that the study is monitored adequately. At each monitoring visit, the monitor will check the best conduct of the study through phone calls with the principal investigator and other study staff. One study initiation visit is planned before the enrolment of the first participant, two on-site monitoring visits during the study implementation, and one close-out visit after the last participant last visit.

#### Adverse event reporting and harms {22}

This is a diagnostic trial that implement PoC tests already available on the market and not an interventional clinical trial testing a new drug/device. Therefore, adverse event is reporting if during the participant follow-up illness get worse or if his/her vital prognosis is threatened. An efficient follow-up visit will be put in place to ensure reporting and management of adverse events requiring medical care on time.

#### Frequency and plans for auditing trial conduct {23}

The study protocol was approved by the national ethics committee for health research and the institutional ethics committee for research in health science of IRSS. The study could be audited at any time by these two authorities, if needed.

#### Plans for communicating important protocol amendments to relevant parties (e.g., trial participants, ethical committees) {25}

All the protocol amendments will be reported to both ethics committees listed above for approbation, as well as the funder and the sponsor. An update will be also poster on the clinical register https://register.clinicaltrials.gov/prs/app/action/SelectProtocol?sid=S000BO17&selectaction=Edit&uid=U0002BK3&ts=2&cx=burdqn.

#### Dissemination plans {31a}

The dissemination of study findings is planned through scientific papers, conferences, meeting with stakeholders and various audience depending to their interest (healthcare workers and local community).

## Discussion

In Sub-Saharan Africa (SSA), acute febrile illnesses are one of the common reasons to seek medical care and are associated with considerable morbidity and mortality [23]. The introduction of malaria rapid diagnostic tests (RDTs) in the routine health care practice at the level of primary health care posts has considerably improved the management of malaria infection and reduced the unnecessary prescriptions of antimalarials. However, there is evidence that this positive development in malaria control program also has a downside due to an increases use of antibiotic in the malaria RDT negative group presenting with fever and this may threat the antibiotic efficacy. Moreover, this situation becomes exacerbated when the malaria RDTs used are still below the expected diagnostic performance in terms of sensitivity and specificity. It is then important to adapt the current practice at primary health facilities in the framework of universal health coverage (UHC) policy adopted by Burkina Faso.

This project has been designed to promote the evidence-based diagnostic for a better management of illnesses in malaria endemic area and reduce the unnecessary prescription of antimicrobials. The project will allow to assess the contribution of an algorithm involving two-step malaria RDT detecting PfHRP2 and pLDH supported by point-of-care tests (PoC tests) for bacterial infection in the management of acute febrile diseases and antimicrobial prescriptions. The project will contribute to (i) save lives by establishing the real cause of fever, (ii) reducing the high cost of treatment during fever episode, due to the inappropriate prescription of antibiotic, (iii) and tackling the antimicrobial resistance (AMR). The project is therefore in line with global effort for the achievement of SDG3 by Horizon 2030.

## Trial status

This is version 02, dated 1 October 2021, of the protocol. Ethical approval has been obtained from the national ethical committee (DELIBERATION N°2021-04-084) and the institutional ethical committee for research in health science of IRSS (N/Réf. A07-2021/CEIRES). Study staff are recruited and trained. At the time of submission, the recruitment of study participants started in 4 March 2022. The trial is ongoing and expected to be completed 28 February 2023 and the final results of the study are expected end of 2023.

## Data Availability

Any data required to support the protocol can be supplied on request.

## Abbreviations

AI: Artificial intelligence
AMR: Antimicrobial resistance
CHU: Centre Hospitalier Universitaire
COVID-19: Coronavirus disease 2019
CRF: Case report form
CRFs: Case record forms
CRP: C-reactive protein
CRUN: Clinical Research Unit of Nanoro
e-Algorithm: Electronic algorithm
e-CRF: Electronic case report form
EDCTP: European & Developing Countries Clinical Trials Partnership
GDT: Guideline of diagnostic and treatment
HIV: Human immunodeficiency virus
HRP2: Histidine-rich protein
IMCI: Integrative management of childhood illnesses
IRSS: Institut de Recherche en Sciences de la Santé
IRSS-DRCO: IRSS-Direction Régional du Centre Ouest
LMICs: Low- and middle- income countries
M6: Month 6
MoH: Ministry of Health
PCR: Polymerase chain reaction
PfHRP2: Plasmodium falciparum histidine-rich proteins
pLDH: Parasite lactate dehydrogenase
PoC: Point-of-care
RDT: Rapid diagnostic test
RDT-DA: RDT decisional algorithm
RDTs: Rapid diagnostic tests
SDG3: Sustainable goal
SPSS: Statistical Package for the Social Sciences
SSA: Sub-Saharan Africa
UHC: Universal health coverage
WBC: White blood cell
WHO: World Health Organization
